# Development and Implementation of Situational Judgment Tests as an Evaluation Method for Training Oncology Physicians: Application in the KOKON-KTO Training

**DOI:** 10.1007/s13187-021-01973-9

**Published:** 2021-03-02

**Authors:** Alizé A. Rogge, Stefanie M. Helmer, Claudia Kiessling, Claudia M. Witt

**Affiliations:** 1 Institute for Social Medicine, Epidemiology, and Health Economics, Charité – Universitätsmedizin Berlin, corporate member of Freie Universität Berlin, Humboldt-Universität zu Berlin, and Berlin Institute of Health, Berlin, Germany; 2 Department of Psychosomatic Medicine, Center for Internal Medicine and Dermatology, Charité - Universitätsmedizin Berlin, corporate member of Freie Universität Berlin, Humboldt-Universität zu Berlin, and Berlin Institute of Health, Berlin, Germany; 3 Institute for Health and Nursing Science, Charité – Universitätsmedizin Berlin, corporate member of Freie Universität Berlin, Humboldt-Universität zu Berlin, and Berlin Institute of Health, Berlin, Germany; 4 Chair for Personal and Professional Development in Health Profession Education, Faculty for Health, Department for Human Medicine, University of Witten/Herdecke, Witten, Germany; 5 Institute for Complementary and Integrative Medicine, University of Zurich and University Hospital Zurich, Sonneggstrasse 6, 8091 Zurich, Switzerland; 6 Center for Integrative Medicine, University of Maryland School of Medicine, Baltimore, MD USA

**Keywords:** Communication skills, Web-based assessment, Medical education, Situational judgment test, E-learning

## Abstract

Situational judgment tests (SJTs) are often used in aptitude testing and present practice-specific challenges. Their implementation into online training programs provides the opportunity to assess learning progress and improve training quality. In this study, text-based SJTs for oncology physicians were developed, validated, and implemented into the KOKON-KTO training which uses a blended learning training format to teach oncology physicians how to consult cancer patients on complementary and integrative medicine (CIM). The SJT was implemented to measure the e-learning results. In the development and validation phase, a total of 15 SJTs (each SJT including 1 best choice answer based on training content and 4 distractors; 9 SJTs for oncologists and 6 SJTs for oncology gynecologists only) were developed by an interprofessional team (*n*=5) using real-case vignettes and applying an in-depth review process. Best answers were validated by experts (oncologists and oncology gynecologists) with experience in advising cancer patients on CIM. In the implementation and evaluation phase, SJTs were answered by KOKON-KTO training participants (*n*=19) pre- and post e-learning. Results were analyzed using descriptive measurements, item difficulties, and Cohen’s *d* for effect size pre- and post-training. The experts (*n*=12, 49.8% gynecologists) agreed with best choice answers (69.4% for oncology gynecology; 81.5% for oncology) in 12 out of 15 SJTs. Comparing pre- and post-training scores, KOKON-KTO training participants were able to improve knowledge substantially (effect sizes for oncologists *d*=1.7; oncology gynecologists *d*= .71). Future studies need to increase the number of experts and SJTs in order to apply further psychometric measurements. As part of the KOKON-KTO study, this project is registered as DRKS00012704 on the “German Clinical Trials Register” (Date of registration: 28.08.2017).

## Introduction

Digital learning in medicine is on the advance with rising numbers for e-learning courses [1–4]. Online training approaches provide the advantages of time flexibility, geographical independence, and access to a multitude of different resources [5]. Deep learning and skills improvement can be enhanced by using different digital learning methods such as virtual reality or film sequences of practice specific work scenarios [6, 7]. However, participants’ learning progress as part of the training evaluation is often not easy to examine, and no consensus has yet been established on assessment methods as gold standard [8].

According to Kirckpatrick et al. [9], a training program can be evaluated at different outcome levels: reaction (e.g., participants’ satisfaction with the course), learning (e.g., increased knowledge), behavior (e.g., demonstrated change of behavior in a performance-based testing), and impact (improvement of patient care). The higher the level, the more difficult it becomes to assess the outcome. Learning progress and thereby the effect of a training program can be evaluated by self-assessment and different forms of objective assessments.

To reach a higher degree of external validity in training, assessments should be developed as closely as possible to practice specific work scenarios, and hence test skills necessary for daily work. This may be enhanced by using context stimuli within the assessments to activate higher order cognitive processes such as systematic decision-making [10].

A situational judgment test (SJT) is often used in aptitude testing as part of contextual competence diagnostics [11], and may be used in employment procedures to detect professional behavior in practice-specific work scenarios. SJTs are usually presented in a written format asking to select one answer out of a given selection of options for a dilemma in a relevant work scenario [11, 12]. The most appropriate answer (best choice answer) is often predefined and may represent a theoretical model validated through expert opinions (also known as theory-based answer) [13]. As SJTs may be presented in the form of multiple-choice questions, low financial and personnel resources are required after the development and evaluation in comparison to other contextual learning methods such as consultations with standardized patients. Testing on both, situational awareness and medical knowledge, the SJTs may bridge the gap between practical work-specific and academic cognitive–based skills. SJTs have been used as a valid method of selection for medical students in the past. However, no broad implementation of the SJTs in postgraduate physician training and in a form of an e-learning training has yet been conducted [14–17].

This study aims to develop and validate SJTs for oncology physicians who are trained in advising their cancer patients on complementary and integrative medicine (CIM) [18]. As a next step, the SJTs were implemented into the KOKON-KTO training as part of an e-learning intervention and used as a method to evaluate the training [19]. The KOKON-KTO training uses a blended learning format (e-learning and skills-training workshop) to train oncology physicians how to consult cancer patients on complementary and integrative medicine during their cancer treatment [18].

## Methods

### Development and Validation of the SJTs

#### Development Process

SJTs were structured by first presenting the patient’s cancer history, followed by individual patient information (age, family status, reason for CIM consultation), and a lead-in question asking the participant to react to the patients’ CIM concern by choosing one out of five given options (single best-response format).

#### SJT Questions

An interprofessional team (*n*=5; oncologist, educational researcher, psychologist, public health researcher, medical doctor specialized in CIM) developed SJTs based on real-case vignettes of cancer patients wishing to receive CIM advice by their treating oncology physician. Using real-case vignettes of cancer patients interested in CIM, we aimed to develop SJTs on relevant, practice-specific work scenarios. SJTs covered cancer entities for oncology physicians (lung cancer, colon cancer, pancreatic cancer) and for oncology gynecologists (breast cancer and ovarian cancer). For each cancer entity, three SJTs were developed which resulted in 15 SJTs in total.

#### SJT Answers

For each question, the best choice answer was derived from the KOKON-KTO consultation manual [18] taking into account the competencies for Integrative Oncology [20]. To add suitable and realistic distractors, two physicians (one oncology gynecologist and one medical oncologist; both regularly advising cancer patients on CIM), as well as two experienced CIM researchers (psychologist, public health researcher), each added one answer to the SJTs, resulting in five answers per SJT (four distractors, one best choice answer according to KOKON-KTO consultation manual).

**Fig. 1:**
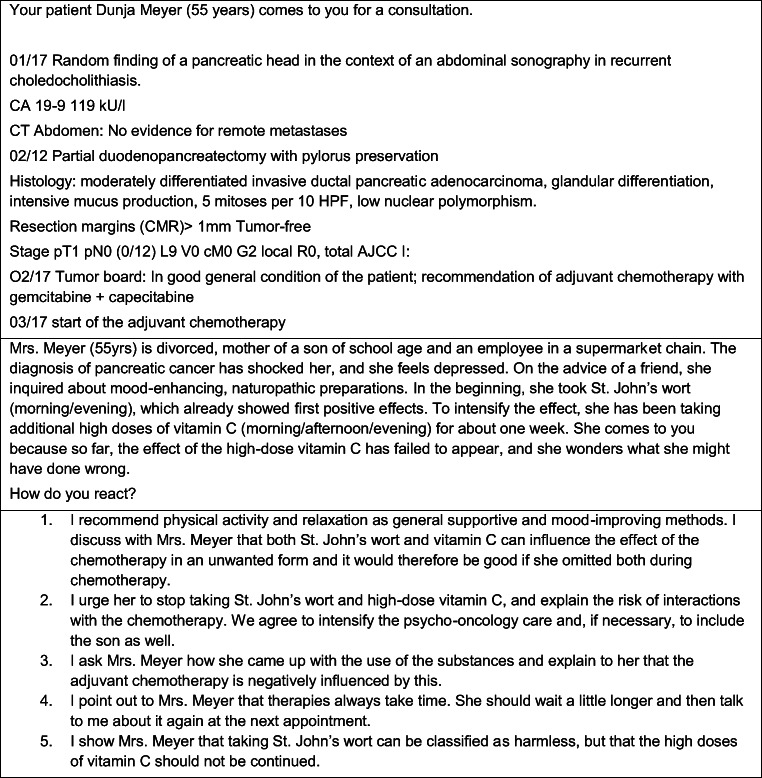
SJT example for oncology

An in-depth review process using inductive and deductive coding strategies in the development team was conducted. Three SJTs per cancer entity were developed. An example for an SJT is given in Fig. 1. Before starting the case vignette process, all names of the case vignette patients were changed and patient’s history was altered so that it cannot be traced back to individuals.

#### Validation Process

SJTs were validated by using an expert panel of oncology physicians with experience in consulting cancer patients on CIM. The experts received SJTs in an anonymous online survey and were asked to indicate the SJT answer most suitable to the way they would answer this question in their clinical routine. The SJT answer must reach more than 50% of expert agreement in order to be accepted by the expert panel. Moreover, experts were able to give additional information on the structure of SJTs at the end of the survey.

#### Experts in the Field

Oncology physicians were eligible if they had previous evidence-based CIM training and worked with cancer patients. Experts were contacted via e-mail and recommended from members of the KOKON-KTO steering board [21] due to (I) their CIM experience in oncology or (II) their participation in a previous study teaching oncology physicians how to advice cancer patients on CIM [22]. Each expert filled out a baseline questionnaire and provided data on gender, specialization, age, and experience in oncology (in years).

### Implementation of SJTs and Effectiveness of the KOKON-KTO Training

#### Implementation Process

The SJTs were incorporated into the e-learning intervention of the KOKON-KTO training [18]. The e-learning was developed according to learning objectives following constructive alignment rules [23] and the underlying consultation manual of the training [18], as well as international educational competencies for health professionals working in integrative oncology [20].

#### Evaluation

SJTs were used before and after the e-learning aiming to assess participants learning progress, and hence the effectiveness of the training. In a single best choice format, participants were asked to choose one out of five answers for each SJT, meaning to choose the answer that best corresponded to their own action in the described situation before and after the e-learning. No time limit was set for completion of SJTs once started.

#### Participant Sample

Oncology physicians (50% oncology and 50% oncology gynecology) participated in the KOKON-KTO study [24], and received the e-learning intervention including the SJTs as part of the study procedure. They were specialized in the field of oncology, and stated to have little CIM knowledge and experience in advising cancer patients on CIM before receiving the KOKON-KTO training. Participants included in the study provided written informed consent to the study team. Participation was on a voluntary basis; however, oncology physicians received 34 CME points by the German Physician Association after the successful completion of the KOKON-KTO training [18]. Baseline data such as gender, medical specialization, age, and experience in oncology were collected as part of the KOKON-KTO study [24].

### Data Analyses

Descriptive statistics were used for the SJTs and results are displayed as total scores standard deviation (SD) and percentages. Then, the highest total score for oncology SJTs was 9 and for oncology gynecology SJTs 6 (1 point per correctly answered SJT).

In the development and implementation phase, item difficulties were assessed for the description of item and test characteristics (dividing the number participants answering the SJT correctly by the total number of participants answering the SJT). In case participants’ answers post e-learning were congruent with experts’ answers and best choice answers, construct validity was presumed in hypothesizing that skilled participants would score higher in the test than unskilled participants.

Moreover, in the implementation phase, the average total test score was calculated for the overall group, and the Pearson correlation was conducted in order to analyze a possible link between years working with cancer patients and the achieved test score in the SJTs. Effect sizes were calculated per specialization (medical oncology, oncology gynecology) for the change between the pre- and post-training measurements (Cohen’s *d*).

## Results

### Development of the SJTs

An example for an SJT is given in Fig. 1 (interested readers are welcome to contact the corresponding author for further information on the developed SJT).

#### Validation of the SJTs by the Expert Group

A total of 40 oncology physicians received an invitation to the online survey of which 12 completed the SJTs (female: 66.7%). Approximately, 50% of experts worked in the field of oncology (general oncology: 16.7%, gastrointestinal or endocrine cancer: 16.7%, ear-nose-throat: 16.7%, internal medicine and hematology: 49.9%) and around 50% of experts worked in the field of oncology gynecology (breast cancer only: 16.7%, general gynecology and palliative care: 83.3%). The experts’ mean age was 48.0 (SD ± 10.2), and the average years of experience working with cancer patients was 17.7 (SD ± 11.0). About one-third of experts (33.3%) stated to have little experience in giving CIM advice regularly in their daily work.

The majority of experts agreed with the theory-based answer deriving from the underlying KOKON-KTO consultation manual for the KOKON-KTO training [18] (see Table 1 for expert test scores).

**Table 1:** Expert test scores in the SJT

SJT*	Medical oncology (*n*=6)*n* (%)	Oncology gynecology (*n*=6)*n* (%)	Item difficulty (%)
Item 1		6(100)	1.0
Item 2		3(50)	.5
Item 3		6(100)	1.0
Item 4		2(33.3)	.33
Item 5		3(50)	.5
Item 6		5(83.3)	.83
Item 7	4(66.7)		.67
Item 8	5(83.3)		.83
Item 9	4(66.7)		.67
Item 10	4(66.7)		.67
Item 11	6(100)		1.0
Item 12	6(100)		1.0
Item 13	6(100)		1.0
Item 14	5(83.3)		.83
Item 15	4(66.7)		.67

*Congruent with best-choice answer

On average, oncology gynecologists reached 4.1 of 6 points (SD ± 1.2; 69.4%) as total test score for their SJTs, and medical oncologists 7.3 of 9 points (SD ± 1.5; 81.1%) as total test score. No significant correlation between years of working with cancer patients and test scores for oncology gynecologists (*r* = .21) and for oncologists (*r* = −.005) was detected.

In the open-ended answers, two experts stated that they found it difficult to choose one answer as many options seemed reasonable and one found that not all possible scenarios were presented.

### Implementation of SJTs and Effectiveness of the KOKON-KTO Training

Both groups of oncology physicians were able to increase their performance in the pre-post comparison (see Table 2).

**Table 2:** Results of the SJTs pre- and post-KOKON-KTO training

	SJT*	Medical oncologists (*n*=12)*n* (%)	Oncology gynecologists (*n*=11)*n* (%)	Item difficulty
Pre-training	Item 1		5(45.5)	.46
	Item 2		6(54.5)	.55
	Item 3		5(45.5)	.46
	Item 4		3(25.0)	.25
	Item 5		7(63.6)	.64
	Item 6		5(45.5)	.46
	Item 7	1(8.3)		.08
	Item 8	8(66.7)		.67
	Item 9	5(41.7)		.42
	Item 10	2(16.6)		.17
	Item 11	6(50)		.50
	Item 12	7(58.3)		.58
	Item 13	9(75)		.75
	Item 14	3(25)		.25
	Item 15	6(50)		.50
		Medical oncologists (*n*=7)*n* (%)	Oncology gynecologists (*n*=8)*n* (%)	
Post-training	Item 1		4(50)	.50
	Item 2		4(50)	.50
	Item 3		6(75)	.75
	Item 4		6(75)	.75
	Item 5		5(62.5)	.63
	Item 6		7(87.5)	.88
	Item 7	3(42.9)		.43
	Item 8	7(100)		1.0
	Item 9	7(100)		1.0
	Item 10	6(85.7)		.86
	Item 11	7(100)		1.0
	Item 12	7(100)		1.0
	Item 13	3(42.9)		.43
	Item 14	4(57.1)		.57
	Item 15	7(100)		1.0

**Fig. 2:**
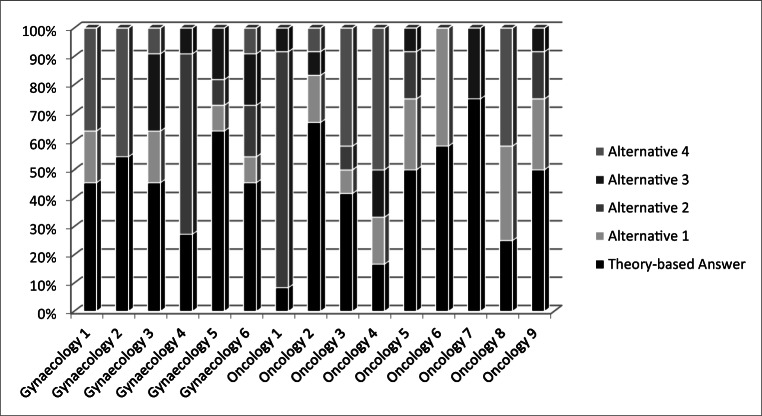
Distribution of SJT results at baseline

**Fig. 3:**
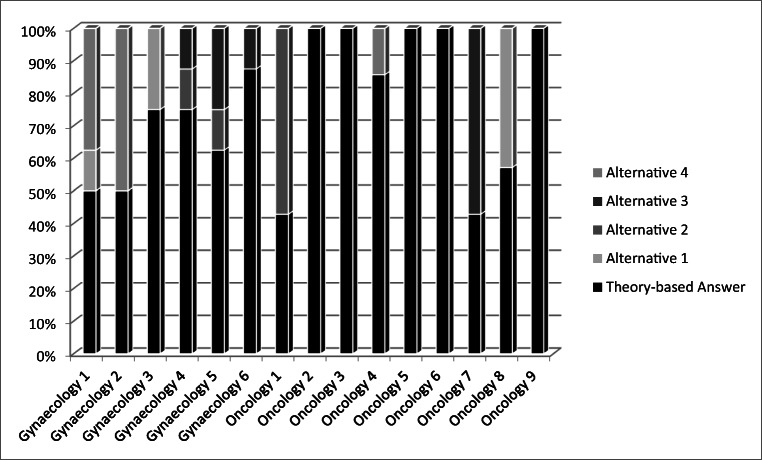
Distribution of SJT results after the training

For the oncology gynecologists, the pre-training total score in *n*=11 was 2.8 out of 6 (SD ± 1.7; 46.6%) and the post-training score in *n*=8 was 4 out of 6 (SD ± 1.1; 66.7%; *d*=.71). For the medical oncologists, the pre-training total score in *n*=12 was 3.9 out of 9 (SD ± 2.0; 43.5%) and the post-training score in *n* = 7 was 7.3 out of 9 (SD ± 1.3; 81.0%; *d*=1.7) (see Figs. 2 and 3 for distribution of SJTs results at baseline and after the training).

## Discussion

This project resulted in the development of 15 SJTs with 6 SJTs specifically developed for oncology gynecology and 9 SJTs for medical oncologists. The SJTs were validated by experts in the field, and later implemented in the KOKON-KTO training [18] to evaluate trainings effects. When comparing the results of the SJTs before and after the KOKON-KTO training, participants showed improvements.

Using a systematic approach as performed in previous studies [25, 26], the 15 SJTs took into account the educational competencies of the respective field of cancer care [20] and were developed involving an international and interprofessional development team. Furthermore, the SJTs were then validated by oncology gynecologists and medical oncologists working in the field. Considering the results of the validation process, in 80% of SJTS (12 out of 15), the best answer based on the KOKON-KTO manual was accepted by the experts in the field. Hence, it can be assumed that most of the developed SJTs (80%) and their best answers provided a realistic picture of the practical work.

The SJTs were answered before and after the KOKON-KTO training [18]. Considering the pre- and post-comparison of the SJT results, training participants were able to increase their knowledge. Due to the small number of participants, we decided to present only findings from descriptive analysis. However, the effect sizes indicate that the training had positive effects on the participants’ knowledge to give CIM advice to cancer patients. Comparing the results from the experts and the results from the training participants after the KOKON-KTO training, it seems the KOKON-KTO training enabled participants to reach similar results as experts in the field when consulting cancer patients on CIM. SJTs might be especially useful when investigating on-the-job behaviors in medicine. In a recent meta-analysis, the use of SJTs as a selection method in medicine for under- and postgraduate was supported by evidence [27]; however, there is still little evidence for the use of SJTs in the evaluation of training programs [28] and hence, its usage has yet to be supported by further studies.

This project has further limitations. In the validation phase, some experts stated difficulties in choosing only one of the given answers as many seemed reasonable, or not all possible scenarios were displayed. Since the SJTs are based on the KOKON-KTO consultation manual [18], which gives clear advice and recommendations on how to give CIM consultations, we chose a single best choice answer for the development of the SJTs. As discussed in a previous study [29], difficulties occur when SJTs are only based on academic curricula that, unfortunately, not always represent the everyday practice in a medical setting. However, it is up to discussions if more solutions to one problem might be possible with greater experience in giving CIM consultations. Further studies should test different answer options such as rank order or rating scales to meet the needs of the experts explained above [30].

Moreover, due to the expert recruitment method (recommendation of participants), response bias cannot be ruled out when interpretating the study results [30]. The small number of SJTs per expert and participant was determined to avoid fatigue and to ensure participation. Due to the small number of SJTs and the sample size of experts, we decided not to calculate Cronbach’s alpha for internal consistency of the SJTs and further methods of test theory such as criterion and criterium validity. Our focus was on feasibility and credibility of the chosen assessment method and to do first steps in developing and validating SJTS in the field of CIM. Comparing the number of SJTs developed for the KOKON-KTO training to existing programs such as the Association of American Medical colleges SJT exam [31] providing 24 SJTs to support admission officers to assess professionalism in medical schools, further investigations on the small set of developed SJTs with methods of the classical test theory were not seen reasonable. Additionally, more oncology physicians completed the SJTs before than after the training, but because of the anonymous nature of the survey, reasons for this remain unclear.

As we know of, this is the first time SJTs have been used as a method of evaluation in oncology physicians postgraduate training. To ensure quality of further postgraduate and continuous education programs and trainings, SJTs might be an economical and an easy way to implement an assessment tool to detect participants overall learning progress. As presented in the study, SJTs might be useful not just as a selection method (e.g., display of performance) but also as an assessment tool for evaluations (e.g., for training curricula). In future studies, the presentation of the developed SJTs may be varied to detect possible adherence factors such as design features [32, 33].

## Data Availability

The datasets analyzed during the current study and codes used are available from the corresponding author on reasonable request.

## References

[1] Dunleavy G (2019). Mobile digital education for health professions: systematic review and meta-analysis by the Digital Health Education Collaboration. J Med Internet Res.

[2] Ruiz JG, Mintzer MJ, Leipzig RM (2006). The impact of E-learning in medical education. Acad Med.

[3] Taveira-Gomes T (2016). What are we looking for in computer-based learning interventions in medical education? A systematic review. J Med Internet Res.

[4] Tang B (2018). Online lectures in undergraduate medical education: scoping review. JMIR Med Educ.

[5] Choules AP (2007). The use of elearning in medical education: a review of the current situation. Postgrad Med J.

[6] Hahne AK (2005). Attitude towards computer-based learning: determinants as revealed by a controlled interventional study. Med Educ.

[7] Masiello I, Ramberg R, Lonka K (2005). Learning in a web-based system in medical education. Med Teach.

[8] de Leeuw R (2019). How we evaluate postgraduate medical e-learning: systematic review. JMIR Med Educ.

[9] Kirkpatrick DL, Kirckpatrick JD (2006). Evaluating training programs: the four levels.

[10] Schuwirth LWT, van der Vleuten CPM (2003). ABC of learning and teaching in medicine: written assessment. BMJ.

[11] McDaniel MA (2001). Predicting job performance using situational judgment tests: a clarification of the literature. J Appl Psychol.

[12] Oostrom JK, De Soete B, Lievens F (2015). Situational judgment testing: A review and some new developments.

[13] Lievens F, Peeters H, Schollaert E (2008). Situational judgment tests: a review of recent research. Pers Rev.

[14] Husbands A (2015). Evaluating the validity of an integrity-based situational judgement test for medical school admissions. BMC Med Educ.

[15] Smith DT, Tiffin PA (2018). Evaluating the validity of the selection measures used for the UK’s foundation medical training programme: a national cohort study. BMJ Open.

[16] Kiessling C (2016). Development and validation of a computer-based situational judgement test to assess medical students’ communication skills in the field of shared decision making. Patient Educ Couns.

[17] Schubert S (2008). A situational judgement test of professional behaviour: development and validation. Med Teach.

[18] Witt CM (2020). Training oncology physicians to advise their patients on complementary and integrative medicine: an implementation study for a manual-guided consultation. Cancer.

[19] Witt C (2018). Training oncology physicians to advise their patients on complementary and integrative medicine. J Altern Complement Med.

[20] Witt CM et al (2020) Education competencies for integrative oncology—results of a systematic review and an international and interprofessional consensus procedure. J Cancer Educ. 10.1007/s13187-020-01829-8 10.1007/s13187-020-01829-8 PMC7876161 32783117

[21] KOKON, K.K.i.d.O.-. Lenkungsgremium. 2020 [cited 2020 12.02.]; Available from: https://www.kompetenznetz-kokon.de/gremium. Accessed 30 June 2020.

[22] Blodt S (2016). A consultation training program for physicians for communication about complementary medicine with breast cancer patients: a prospective, multi-center, cluster-randomized, mixed-method pilot study. BMC Cancer.

[23] Anderson L, Kratwohl D, Airasian P (2013). A taxonomy for learning, teaching, and assessing: a revision of Bloom’s Taxonomy of educational objectives. Pearson New International Edition.

[24] Helmer SM et al (2019) Evaluation of a blended-learning training concept to train oncology physicians to advise their patients about complementary and integrative medicine (KOKON-KTO): study protocol for a prospective, multi-center, cluster-randomized trial. Trials 20(90) 10.1186/s13063-019-3193-y PMC6352447 30696465

[25] Patterson F (2019). Evaluation of a situational judgement test to develop non-academic skills in pharmacy students. Am J Pharm Educ.

[26] de Leng WE (2018). Integrity situational judgement test for medical school selection: judging ‘what to do’ versus ‘what not to do’. Med Educ.

[27] Webster ES (2020). Situational judgement test validity for selection: a systematic review and meta-analysis. Med Educ.

[28] Hauenstein NMA, Findlay RA, McDonald DP (2010). Using situational judgment tests to assess training effectiveness: lessons learned evaluating military equal opportunity advisor trainees. Mil Psychol.

[29] Simon E (2015). Does a high ranking mean success in the situational judgement test?. Clin Teach.

[30] Jayakumar N, Kawa B, Zhou S (2015). Ranking and situational judgement test. Clin Teach.

[31] Colleges, A.o.A.M. AAMC SJT Exam Time and Testing Conditions. 2020 [cited 2020 27.10]; Available from: https://students-residents.aamc.org/applying-medical-school/article/aamc-sjt-exam-time-and-testing-conditions/. Accessed 15 Oct 2020.

[32] De Leng WE (2018). Influence of response instructions and response format on applicant perceptions of a situational judgement test for medical school selection. BMC Med Educ.

[33] Juster FR (2019). Addressing the diversity-validity dilemma using situational judgment tests. Acad Med.

